# Triplet state harvesting and search for forbidden transition intensity in the nitrogen molecule

**DOI:** 10.3389/fchem.2022.1005684

**Published:** 2022-10-18

**Authors:** B. F Minaev, O. O Panchenko, V. A Minaeva, H Ågren

**Affiliations:** ^1^ Department of chemistry and nanomaterial sciences, Bohdan Khmelnytsky National University, Cherkasy, Ukraine; ^2^ Department of Physics and Astronomy, Uppsala University, Uppsala, Sweden

**Keywords:** triplet–singlet transitions, nitrogen molecule, Vegard–Kaplan band, Wilkinson band, Herzberg I band analog

## Abstract

Triplet excited states of the N_2_ molecule play an important role in electric discharges through air or liquid nitrogen accompanied by various afterglows. In the rarefied upper atmosphere, they produce aurora borealis and participate in other energy-transfer processes connected with atmospheric photochemistry and nightglow. In this work, we present spin–orbit coupling calculations of the intensity of various forbidden transitions, including the prediction of the electric dipole transition moment of the new $1^{3}\Sigma_{g}^{-}\leftarrow {\;A}^{3}\Sigma_{u}^{+}$ band, which is strongly prohibited by the (+|−) selection rule, the new spin-induced magnetic ${B^{\prime }}^{3}\Sigma_{u}^{-}\leftarrow \;A^{3}\Sigma_{u}^{+}$ transition, magnetic and electric quadrupole transitions for the B^3^Π_g_ ${\leftarrow X}^{1}\Sigma_{g}^{+}$ Wilkinson band, and the Lyman–Birge–Hopfield a^1^Π_g_ ← X^1^Σ_g_ transition. Also, two other far-UV singlet–singlet quadrupole transitions are calculated for the first time, namely, the Dressler–Lutz a"^1^Σ_g_^+^–X^1^Σ_g_^+^ and the less studied z^1^Δ_g_–X^1^Σ_g_^+^ weak transitions.

## Introduction

The great flux of solar energy through the upper atmosphere can be harvested by the rarefied gases of molecular and atomic components of the Earth’s mesosphere and lower thermosphere (MLT) regions (Minaev and Panchenko, 2020). The ground states of such abundant O_2_ (^3^Σ_g_^–^), O (^3^P), and N (^4^S) species of MLT possess high multiplicity, and thus their lowest excited states are metastable, having a low electronic spin and strongly forbidden radiative relaxation (Wilkinson and Mulliken, 1959; Brown and Winkle, 1970; Minaev and Panchenko, 2020). Their long-lived emission to the ground state provides the possibility to harvest visible and near-UV solar radiation engaged in various energy transfer processes, which determine the climate, meteorology, and weather conditions (Minaev and Panchenko, 2020). In contrast, the ground state of the nitrogen molecule possesses zero spin and several high-energy triplet excited states with deep potential wells. The lowest of them, $A^{3}\Sigma_{u}^{+}$ , can harvest a stock of 6.22 eV energy, being a strongly metastable triplet state with a relatively long radiative lifetime (τ_r_) of 2 s (Brown and Winkle, 1970; Minaev et al., 1995; Begley et al., 2022). Accounting for the short UV wavelength of the $A^{3}\Sigma_{u}^{+}\to X^{1}\Sigma_{g}^{+}$ transition, this τ_r_ value is indeed unusually large.

**FIGURE 1:**
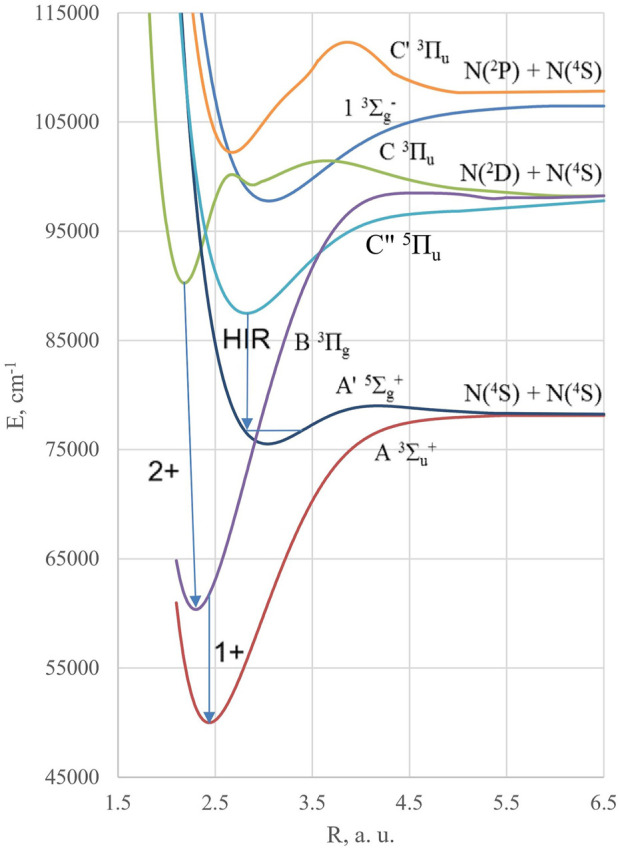
Potential energy curves of several spectroscopy important excited states of the N_2_ molecule. The first (**1+**) and second positive (**2+**) systems are denoted together with the Herman infrared (HIR) emission band.

**FIGURE 2:**
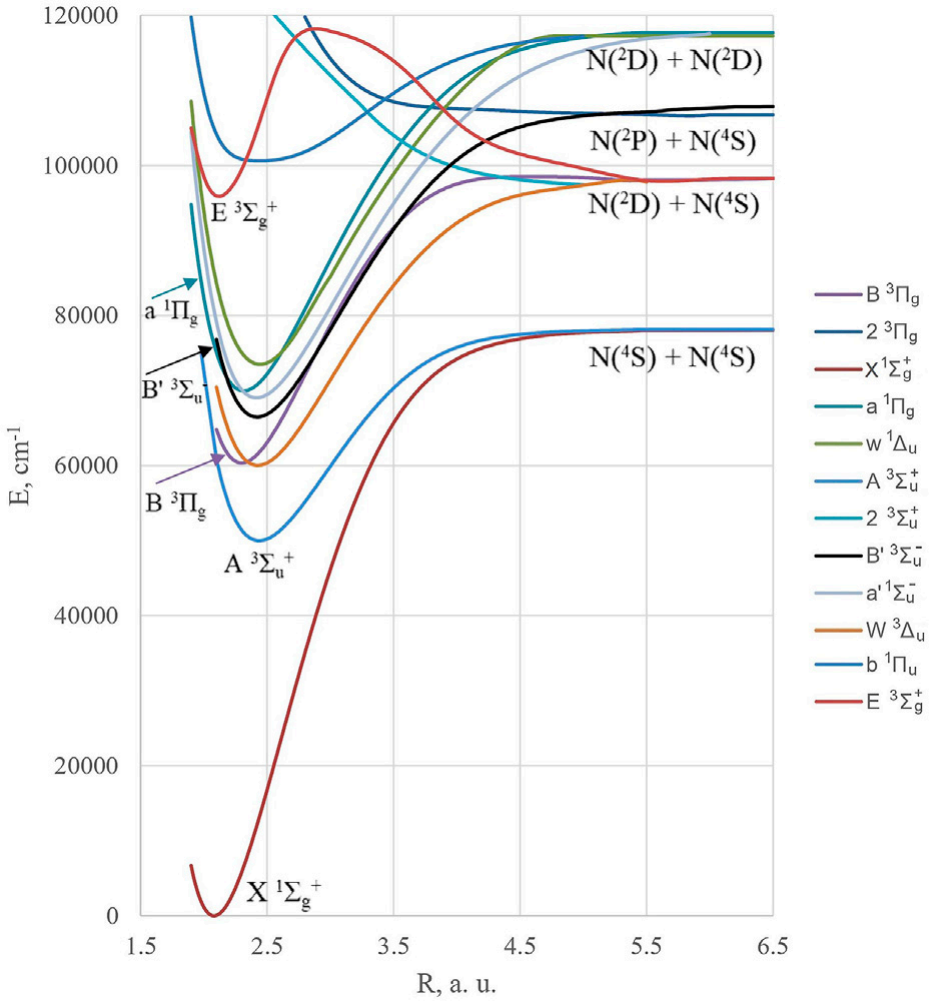
Low-lying bound states, which contribute to the intensity of the studied transition.

N_2_ is a very stable and inert molecule in the ground state XΣ 1g+ with high dissociation energy (D_e_ = 9.76 eV). At the same time, N_2_ possesses a variety of quite stable valence excitations of the π_u_–π_g_ and 3σ_g_–π_g_ types; these excited states have large D_e_ values (around 4–6 eV) and are mostly metastable since their emission to the ground state is strictly forbidden by the electric dipole selection rules (Wilkinson and Mulliken, 1959; Brown and Winkle, 1970; Begley et al., 2022; Minaev et al., 1995; Lewis et al., 2008; Lofthus. and Krupenie, 1977). In gaseous electric discharges, when a molecule is irradiated by an electron flux, N_2_ dissociates into the ground state N (^4^S) atoms; they can recombine forming the lowest singlet (X^1^Σ_g_^+^), triplet (A^3^Σ_u_^+^), and quintet (A′^5^Σ_g_^–^) basic states. The last two, shown in Figure 1, are involved in the so-called active nitrogen phenomenon detected by the characteristic “yellow afterglow” (Brown and Winkle, 1970; Begley et al., 2022). Its study together with aurora borealis involves a large number of metastable states and forbidden transitions in the N_2_ spectrum (Figure 2). The Lewis–Rayleigh afterglow (Brown and Winkle, 1970) in the discharge consists of the first positive system of the nitrogen molecule, extending from IR to the blue edge, being the triplet–triplet B^3^Π_g_ → A^3^Σ_u_^+^ transition (**1+** system) (Lofthus. and Krupenie, 1977). The visible part of the **1+** system was already investigated in 1902 by Deslandres (1902); *ab initio* interpretation of its intensity was achieved by Werner et al. (1984) and a final form by Ni et al. (2017). It should be distinguished from the second positive system of the nitrogen molecule—the C^3^Π_u_ → A^3^Σ_u_^+^ transition (**2+** system) and the infrared Hermann (HIR) band C″^5^Π_u_ → A′^5^Σ_g_^+^ (Figure 1). The main sources of emission of the first and second positive systems in N_2_ discharge are connected with the involvement of the N (^2^D) excited atom into a recombination reaction (Figure 1). The **2+** band system was observed as early as 1869 as it readily appears in ordinary air discharges (Deslandres, 1902), but its rovibronic assignment came much later (Lofthus and Krupenie, 1977). As opposed to the O_2_ molecule (Minaev and Panchenko, 2020), many visible and UV transitions between triplet excited states generated by electric discharge are possible in the nitrogen counterpart (Lofthus. and Krupenie, 1977; Minaev et al., 1995; Lewis et al., 2008). The quintet state A′^5^Σ_g_^+^ and the HIR system of N_2_ have become clear only recently (Partridge et al., 1988; Hochlaf et al., 2010a). They are essentially important for the recombination of the N (^4^S) ground state atoms being the precursor of the Lewis–Rayleigh afterglow. The quintet A′^5^Σ_g_^+^ can predissociate to the B^3^Π_g_ state vibrational levels (*v* = 10–12, Figure 1), though the spin–orbit coupling (SOC) matrix element (ME) <A′^5^Σ_g_^+^|H_so_| B^3^Π_g_> is rather weak near the crossing in order to be efficient for generation of the spontaneous **1+** emission in the recombination of N (^4^S) atoms. At the same time, this SOC ME determines the high radiative probability (Einstein coefficient about 3∙10^4^ s^−1^) of the newly predicted A′^5^Σ_g_^+^ → A^3^Σ_u_^+^ (0–6) transition, which borrows intensity from the **1+** system, as well as from the HIR band (Minaev et al., 2022). The latter source is attributed to a strong SOC between the A^3^Σ_u_^+^ and C″^5^Π_u_ states.

The excited metastable N (^2^D) and N (^2^P) atoms with energies of 2.4 eV and 3.6 eV above the N (^4^S) ground state, respectively (Figure 1), are present with low concentration in the discharge. Their recombination leads to a huge number of excited N_2_ states with varying degrees of stability and spontaneous emission probabilities (Lofthus. and Krupenie, 1977). Several other important states of nitrogen are shown in Figure 2.

## Energy harvesting by triplet states of nitrogen

The triplet excited manifold of the N_2_ molecule is well studied in far-UV absorption and emission spectra (Deslandres, 1902; Lofthus. and Krupenie, 1977; Werner et al., 1984; Partridge et al., 1988; Piper, 1993; Minaev et al., 1995; Lewis et al., 2008; Ndome et al., 2008; Hochlaf et al., 2010a; Ni et al., 2017). In 1932, Vegard detected 120 weak bands in the red-degraded phosphorescence of solid nitrogen through the wide region of 670–170 nm (Lofthus and Krupenie, 1977). Soon after, Kaplan observed similar bands in an N_2_ laboratory discharge (Minaev et al., 1995). The weak Vegard–Kaplan (VK) system was first detected by Wilkinson as absorption bands in a long-path spectrometer at 169 and 128 nm for highly excited vibronic levels (v’ = 6,7) (Wilkinson and Mulliken, 1959). Later on, the VK rovibronic intensity alternations were measured and analyzed very carefully (Lofthus and Krupenie, 1977; Piper, 1993) including *ab initio* calculations for the VK transition probability and many other inter-combination systems (Minaev et al., 1995). SOC calculations within the quadratic response theory (Minaev et al., 1995) explained why the Ogawa–Tanaka–Wilkinson system B′Σ 3u−←XΣ 1g+ is much more intense (70 times) than the Vegard–Kaplan AΣ 3u+←XΣ 1g+ absorption and why the Tanaka transition CΠ 3u←XΣ 1g+ is the most intense among all known triplet–singlet (T–S) absorption bands at that time (Lofthus and Krupenie, 1977; Minaev et al., 1995). The new T ← S transition DΣ 3u+←XΣ 1g + in the far-UV region predicted by Minaev et al. (1995) was later detected and analyzed by Lewis et al. (2008). The upper DΣ 3u+ state has been observed earlier in the pure nitrogen condensed discharge afterglow through the DΣ 3u+ →B^3^Π_g_ (0, v’‘) emission, which is now known as the fourth positive system (Lofthus and Krupenie, 1977; Minaev et al., 1995). The upper DΣ 3u+ state was shown to be of Rydberg type (Minaev et al., 1995) converging to the ground state N_2_^+^ ion. At longer N–N distances, it avoids crossing with the bound Rydberg state and the valence 3^3^Σ_u_^+^ state potential energy curve (PEC), demonstrating a repulsive character (Minaev et al., 1995). All theoretical predictions of the inter-combination D ← X transition (Minaev et al., 1995) have mainly been supported by later experiments (Lewis et al., 2008). The predicted 0–0 transition is rather intense (f = 2×10^−5^), being the strongest inter-combination of a nitrogen molecule in agreement with measurements (Lofthus and Krupenie, 1977; Lewis et al., 2008). This far-UV region in N_2_ absorption is very dense, being covered by allowed transitions (b^1^Π_u_–X, for example) (Lofthus and Krupenie, 1977), but the D–X (0, 0) band has a clear location in a fortuitous region of the b^1^Π_u_ ← X allowed spectrum, just above its (4,0) band head, enabling the D–X (0, 0) observation (Lewis et al., 2008). All three sublevels of the triplet D state provide four rotational branches in agreement with Minaev et al. (1995), according to rotational and parity selection rules of Hund’s case “b” (Lewis et al., 2008). The small negative zero-field splitting (*λ* = −0.036 cm^−1^ (Lewis et al., 2008)) of the D Σ 3u+ state is in agreement with SOC and spin–spin coupling calculations (*λ* = −0.041 cm^−1^) within the response approach (Loboda et al., 2003; Hochlaf et al., 2010b; Qin et al., 2019; Minaev et al., 2022).

Thus, almost all important singlet–triplet transitions in the molecular nitrogen absorption spectra (up to the far-UV region) from the ground state XΣ 1g+ to the triplet states of the “ungerade” symmetry—the A Σ 3u+,B′Σ 3u− , *W* ^3^Δ_u_, *C* ^3^Π_u_, and *D* Σ 3u+ states—have been calculated by the quadratic response theory within the multi-configuration approach (Minaev et al., 1995), giving results that are in good agreement with experimental intensity distributions (Lofthus. and Krupenie, 1977; Piper, 1993; Lewis et al., 2008). The present work aims to calculate new forbidden transitions in the nitrogen spectra which have not been observed so far but can influence the triplet state harvesting and total kinetic balance of the upper atmosphere.

The B^3^Π_g_ state produced by the second and fourth positive systems (Lofthus and Krupenie, 1977) can further generate **1+** bands, and the lowest triplet AΣ 3u+ state by the cascade in the positive column of electric discharge. We have to note that the B^3^Π_g_ → XΣ 1g+ phosphorescence was not calculated in Minaev et al. (1995), since even an account of SOC cannot overcome its parity prohibition in terms of electric dipole selection rules. The calculation of this transition intensity is an aim of the present work.

The VK transition satisfies the orbital electric dipole selection rule (EDSR) (Minaev et al., 1995), but being spin-forbidden it cannot be effectively induced by direct UV absorption. Thus, the N_2_ (A) state is primarily populated by collisions—in laboratory discharge and the upper atmosphere, this is accomplished through the electron impact and the cascade in the first positive system. The relatively long radiative lifetime enables N_2_ (A Σ 3u+ ) to participate in collisions with the main background gases of the MLT region and to produce chemical reactions with N_2_, O_2_, N, and O species. In particular, the reactions

$$N_{2}\left(A^{3}\Sigma_{u}^{+})+O{(}^{3}P\right)=NO\left(X^{2}\Pi \right)+N^{2}\left(D\right)$$

$$N_{2}\left(A^{3}\Sigma_{u}^{+})+O{(}^{3}P\right)=N_{2}\left(X^{1}\Sigma_{g}^{+})+O{(}^{1}S\right)$$

are the most important ones (Yonker and Bailey, 2019). A recent steady-state MLT model developed for the N_2_ (A Σ 3u+ ) vibrational distribution in the terrestrial atmosphere is supported by comparison with the Vegard–Kaplan dayglow emission from atmospheric photochemistry and ionospheric spectroscopy measurements (Yonker and Bailey, 2019). The steady-state N_2_ (A Σ 3u+,v ) vibrational distribution in the MLT region is found to be shifted to higher (v > 6) levels. This is in agreement with the VK absorption (Minaev et al., 1995) and is important for our study. Direct excitation from the ground N_2_(X) state by the electron impact provides an essential contribution to populating the N_2_ (A, v > 6) sublevels, though their dominant excitation mechanism is the radiative cascade *via* the **1+** system (Bruna and Grein, 2009; Yonker and Bailey, 2019; Ajello et al., 2020). The efficiency of this cascade depends on the B^3^Π_g_ $\to X^{1}\Sigma_{g}^{+}$ transition intensity, which in turn is determined by the EDSR-forbidden a^1^Π_g_ $\to X^{1}\Sigma_{g}^{+}$ magnetic-dipole-allowed band system. Intensity calculations of these strongly forbidden transitions are also the purpose of our work.

The N_2_ molecule, the most common and abundant component of the air, plays a crucial role in many high-energy photochemical processes caused by solar radiation in the upper atmosphere (Yonker and Bailey, 2019; Ajello et al., 2020). The discovery of new N_2_ transitions forbidden by the spin-selection rule and induced by SOC perturbation is an important part of optical nitrogen monitoring at different altitudes. The intensity origin of the known emission bands that are forbidden by the electric dipole selection rules is also an important task of N_2_ spectroscopy (Deslandres, 1902; Wilkinson and Mulliken, 1959; Brown and Winkle, 1970; Lofthus and Krupenie, 1977; Werner et al., 1984; Partridge et al., 1988; Piper, 1993; Minaev et al., 1995; Lewis et al., 2008; Ndome et al., 2008; Hochlaf et al., 2010a; Ni et al., 2017; Begley et al., 2022). This work presents multi-reference configuration interaction (MRCI) calculations of the highly excited states of the nitrogen molecule and an explanation of the intensity origin of several forbidden optical transitions. With this aim and background, we have predicted the electric dipole transition moment (EDTM) of the unknown forbidden transition $1^{3}\Sigma_{g}^{-}-A^{3}\Sigma_{u}^{+}$ and calculated its dependence on the internuclear distance. This is a triplet–triplet (T–T) band, the intensity of which is entirely determined by spin–orbit coupling perturbations between various spin sublevels of the T states as was preliminarily shown in a recent work (Minaev et al., 2022). The upper $1^{3}\Sigma_{g}^{-}$ state was earlier calculated by similar MRCI methods (Hochlaf et al., 2010b; Qin et al., 2019), but no experimental manifestations of its existence have been evidenced so far, although the $1^{3}\Sigma_{g}^{-}$ state is predicted with a deep minimum (*D*_*e*_ = 1.23 eV) and high energy above the ground state (*T*_*e*_ = 12.15 eV) (Qin et al., 2019). We believe that the $1^{3}\Sigma_{g}^{-}$ state can be produced by N (^2^P) + N (^4^S) recombination (Figure 1), and that its low vibrational levels can avoid pre-dissociation at low pressure. The N_2_ $\left(1^{3}\Sigma_{g}^{-}\right)$ state possesses a potential energy well located outside the Franck–Condon (FC) region, which is accessible from the metastable $A^{3}\Sigma_{u}^{+}$ state as well as from the ground state. This explains the difficulties with the observation of the corresponding absorption bands. Under these conditions, the emissive $1^{3}\Sigma_{g}^{-}\to \;A^{3}\Sigma_{u}^{+}$ transition from the lowest v’ = 0 sublevel will have the maximum FC factor for the v” = 7–8 vibronic levels of the *A* state. We provide evidence for the existence of this new band in the N_2_ molecule by calculating the transition probabilities through an account of SOC in the first order of the perturbation theory and comparing them with other known forbidden transitions to facilitate the validity of such a prediction. This would be a wide band of low intensity in the range of 209–450 nm with an approximate maximum at 328 nm; it is prohibited by the severe selection rule (+) → (–) but is allowed by spin-selection as a T–T transition (Minaev et al., 2022). Its spin-rovibronic structure would be analogous to the well-known Herzberg I band of molecular oxygen $X^{3}\Sigma_{g}^{-}\to \;A^{3}\Sigma_{u}^{+}$ (Herzberg, 1952; Minaev and Muldakhmetov, 1984; Klotz and Peyerimhoff, 1986).

## Intensity borrowing mechanisms of the forbidden $1^{3}\Sigma_{g}^{-}\to A^{3}\Sigma_{u}^{+}$ transition

For planning intensity calculations of the new band in nitrogen, we first take into account the corresponding well-known and intense transitions of the N_2_ molecule, relevant for our purpose. According to SOC selection rules, the new N_2_ band $1^{3}\Sigma_{g}^{-}\to A^{3}\Sigma_{u}^{+}$ can be formed by spin–orbit coupling-induced mixing of the upper state $1^{3}\Sigma_{g}^{-}$ with the $B^{3}\Pi_{g}$ state and by intensity borrowing from the first positive system $A^{3}\Sigma_{u}^{+}-{\;B}^{3}\Pi_{g}$ , see Eq. 1. To include the SOC effect, we have to add the *Ω* quantization, where *Ω* = L_z_ + S_z_ is the *z*-projection of the total electronic angular momentum and L_z_ and S_z_ are orbital and spin angular momenta projections on the molecular axis (Yonker and Bailey, 2019). The SOC operator can mix states with the same *Ω*; the EDTM selection allows transitions according to the rule ΔΩ = 0, ±1:

|1Σ∼g,1−3〉=|1Σg,1−3〉+〈BΠg,13|HSO|1Σg,1−3〉E(1Σg−3)−E(BΠg3)|BΠg,13〉=|1Σg,1−3〉+СB,1|BΠg,13〉,

〈AΣu,0−+3|er|1Σ∼g,1−3〉=CB,1〈AΣu,0−+3|e×|BΠg,13〉, (1)

**FIGURE 3:**
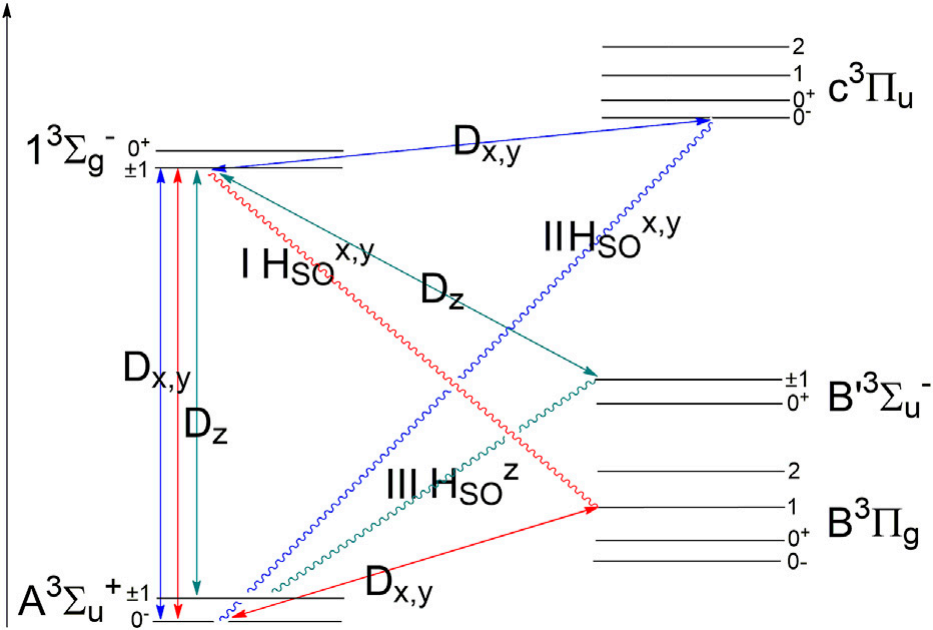
Scheme of intensity borrowing for the forbidden $\Sigma_{g}^{-}\to \;\Sigma_{u}^{+}$ transition. The first and second mechanisms provide perpendicular polarization, and the third one is responsible for the parallel polarization along the N–N axis.

Figure 3 presents this mechanism as the type “I SOC” mixing. By a similar SOC mechanism, the studied forbidden band can borrow EDTM intensity from the newly predicted $1^{3}\Sigma_{g}^{-}\to C^{3}\Pi_{u}$ transition here, Eq. 2; this SOC mechanism of intensity borrowing refers to the “II SOC” type in Figure 3. Both these mechanisms have the perpendicular *x, y* polarization of EDTM; only the *x* component is shown in Eqs. 1–2 for one sublevel of the degenerate Π states (Qin et al., 2019; Minaev et al., 2022).

|AΣ∼u,0−+3〉=|AΣu,0−+3〉+〈CΠu,0−3|HSO|AΣu,0−+3〉E(AΣu+3)−E(CΠu3)|CΠu,0−3〉=|AΣu,0−+3〉+CC,A|CΠu,0−3〉,

〈AΣ∼u,0−+3|er|1Σg,1−3〉=CC,A∗〈CΠu,0−3|e×|1Σg,1−3〉. (2)

Figure 3 provides a good explanation of the relevant intensity sources of the studied $1^{3}\Sigma_{g}^{-}\to \;A^{3}\Sigma_{u}^{+}$ transition, but it would be overloaded if all possible contributions are included. The type “II SOC” mechanism in Figure 3 includes also other states of the C′^3^Π_u_ type (in total five ^3^Π_u_ states are taken into account).

An additional source of intensity borrowing denoted as the type “III SOC” mechanism in Figure 3 includes parallel EDTM for the studied emission band (light polarization along the molecular *z-*axis). By symmetry arguments, the $1^{3}\Sigma_{g}^{-}\to A^{3}\Sigma_{u}^{+}$ transition in N_2_ is similar to the Herzberg I band of the O_2_ molecule, and its probability can be calculated by a similar scheme of intensity borrowing (Minaev and Muldakhmetov, 1984; Klotz and Peyerimhoff, 1986). In the oxygen molecule, the main contribution to the absorption intensity of the Herzberg I band $X^{3}\Sigma_{g}^{-}\to \;A^{3}\Sigma_{u}^{+}$ origins from the SOC mixing between the $A^{3}\Sigma_{u,1}^{+}$ state and the upper term $B^{3}\Sigma_{u,1}^{-}$ of the Schumann–Runge system $X^{3}\Sigma_{g}^{-}\to \;B^{3}\Sigma_{u}^{-}$ (Minaev and Muldakhmetov, 1984), which is the most intense valence transition in molecular oxygen (Herzberg, 1952). This provides a rather unusual (for the ^3^Σ^−^–^3^Σ^+^ band) type of Ω = 1–Ω = 1 parallel transition intensity, though the ΔΩ = 1 selection rule is more typical for such bands with prevailing perpendicular polarization (Herzberg, 1952).

Let us consider the type “III SOC” mechanism in more detail. The SOC-induced mixing between the lowest $A^{3}\Sigma_{u}^{+}$ state and the upper triplet B′Σ 3u− of the Ogawa–Tanaka–Wilkinson system ( ${B^{\prime }}^{3}\Sigma_{u}^{-}-X^{1}\Sigma_{g}^{+})$ can be presented by the perturbation theory in the form:

|AΣ∼u,1+3〉=|AΣu,1+3〉+〈B′Σu,1−3|HSO|AΣu,1+3〉E(AΣu+3)−E(B′Σu−3)|B′Σu,1−3〉=|AΣu,1+3〉+CB′,A|B′Σu,1−3〉. (3)

We can also account for SOC perturbation for the $1^{3}\Sigma_{g}^{-}$ counterpart as follows:

|1Σ∼g,1−3〉=|1Σg,1−3〉+〈EΣg,1+3|HSO|1Σg,1−3〉E(1Σg−3)−E(EΣg−+3)|EΣg,1+3〉=|1Σg,1−3〉+CE,1|EΣg,1+3〉. (4)

The EDTM between the perturbed states (3) and (4) is equal to

〈1Σ∼g,1−3|er|AΣ∼u,1+3〉=CE,1∗〈EΣg,1+3|ez|AΣu,1+3〉+CB′,A〈1Σg,1−3|ez|B′Σu,1+3〉. (5)

This means that the (+|−) forbidden transition $1^{3}\Sigma_{g}^{-}-A^{3}\Sigma_{u}^{+}$ can borrow intensity from ED-allowed $E^{3}\Sigma_{g}^{+}-A^{3}\Sigma_{u}^{+}$ and $1^{3}\Sigma_{g\;}^{-}-{B^{\prime }}^{3}\Sigma_{u}^{-}$ transitions. The latter contribution is a formal symmetry analog of the Schumann–Runge O_2_ transition. The SOC mixing mechanism shown in Eq. 3 is presented in Figure 3 by the intensity borrowing scheme “III-SOC”. The SOC-induced mechanism from Eq. 4 is not shown in Figure 3 to avoid overloading. The SOC matrix element (ME) in Eq. 3 is equal to zero in a semi-empirical approximation with the neglect of differential overlap:

$$H_{SOC}=\underset{A}{\sum }\varsigma_{A}\underset{i}{\sum }{\vec{l}}_{A,i}{\vec{s}}_{i}=\underset{i}{\sum }{\vec{B}}_{i}{\vec{s}}_{i},$$ (6)

where $\varsigma_{A}$ is the SOC constant for the valence shell of the A atom and ${\vec{l}}_{A,i}{\vec{s}}_{i}$ is a scalar product of the orbital and spin operators for the *i*th electron (Minaev and Muldakhmetov, 1984; Minaev et al., 1993). For the pure main configurations of the $A^{3}\Sigma_{u}^{+}$ and ${B\prime }^{3}\Sigma_{u}^{-}$ states, the SOC ME is equal to $\frac{1}{2}\left(B_{\pi_{u,x}\pi_{u,y}}-B_{\pi_{g,x}\pi_{g,y}}\right)$ ; this expression is zero with the neglect of differential overlap since $B_{\pi_{u,x}\pi_{u,y}}=B_{\pi_{g,x}\pi_{g,y}}$ (Minaev and Muldakhmetov, 1984), but the account of overlap in normalization of the $\pi_{u}$ and $\pi_{g}$ molecular orbitals in the r-centroid approach (1.282 Å) leads to the different estimations $B_{\pi_{u,x}\pi_{u,y}}$ = 85 cm^−1^and $B_{\pi_{g,x}\pi_{g,y}}$ = 60 cm^−1^. Thus, the SOC ME in Eq. 3 reaches a non-zero value of 12.5 cm^−1^, which is rather close to the MRCI result. This scrutinized analysis shows the importance of the contribution expressed by Eq. 3 and the analogy with the Herzberg I Schumann–Runge transition coupling in the O_2_ molecule (Minaev and Muldakhmetov, 1984). The denominator in Eq. 3 is rather small and homogeneously changes with *r* distance (Figure 3). Although the $1^{3}\Sigma_{g\;}^{-}-{B^{\prime }}^{3}\Sigma_{u}^{-}$ transition is relatively weak in the N_2_ molecule (EDTM = 0.026 ea_0_ at *r* = 1.4 Å) (Qin et al., 2019), its contribution to the final EDTM of Eqs 1–5 is the largest. The EDTM of the $E^{3}\Sigma_{g}^{+}-A^{3}\Sigma_{u}^{+}$ transition (the Herman–Kaplan band system (Lofthus. and Krupenie, 1977)) has a smaller value (0.017 and 0.0105 ea_0_ at *r* = 1.28 and 1.4 Å, respectively) (Qin et al., 2019), as well as the SOC ME in Eq. 4 at these distances (5.2 cm^−1^) (Hochlaf et al., 2010b).

We have stressed before the EDTM component of the studied intensity borrowing from the $1^{3}\Sigma_{g}^{-}\to B^{\prime }{\;}^{3}\Sigma_{u}^{-}$ band, which is a formal analog of the Schumann–Runge system of oxygen (Minaev and Panchenko, 2020). Thus, we can compare various contributions to the intensity of this so-far unknown transition with the well-known data for O_2_ and N_2_ spectra (Herzberg, 1952; Lofthus. and Krupenie, 1977; Minaev et al., 1995; Lewis et al., 2008). The intensity borrowing contribution from the first positive system $B^{3}\Pi_{g}\to A^{3}\Sigma_{u}^{+}$ in Eq. 1 can be compared with the Vegard–Kaplan S–T transition intensity presented in Eq. 7 (Minaev et al., 1995), which explains an extremely low spontaneous emission of the VK system.

<XΣ 1∼g+|er|AΣ 3∼u+≥=CX,B<BΠ 3g|er|AΣ 3u+>+Cb,A<bΠ 1u|er|X1Σg+>, (7)

CX,B=<XΣ 1g+|Hso|BΠ 3g>E(BΠ 3g)−E(XΣ 1∼g+), Cb,A=<bΠ 1u|Hso|AΣ 3u+>E(AΣ 3u+)−E(bΠ 1u).

As shown in Figure 2, the two denominators in Eq. 7 have opposite signs. The first denominator E(B)–E(X) decreases with *r* distance prolongation, whereas the second one, E(A)–E(b), increases by an absolute value with *r*. In the vicinity of the ground state equilibrium *r*_*e*_ distance (1.098 Å), both contributions tend to cancel each other, and the EDTM value crosses the zero point (Minaev et al., 1995). In the whole FC region, the EDTM is still close to zero, and the VK system has very low intensity both in absorption and emission. Although both the SOC ME values in the nominators of Eq. 7 are rather large (Bruna and Grein, 2009; Hochlaf et al., 2010b) as well as the transition moments of the **1+** and bΠ 1u→X1Σg+ systems (Qin et al., 2019), the cancellation of the two big terms in Eq. 7 is the only reason for the relatively large lifetime of the N_2_ $\left(A^{3}\Sigma_{u}^{+}\right)$ state. To a large extent, this is also the reason for the efficient solar energy harvesting by the triplet states of nitrogen molecules and the aurora borealis phenomena.

**FIGURE 4:**
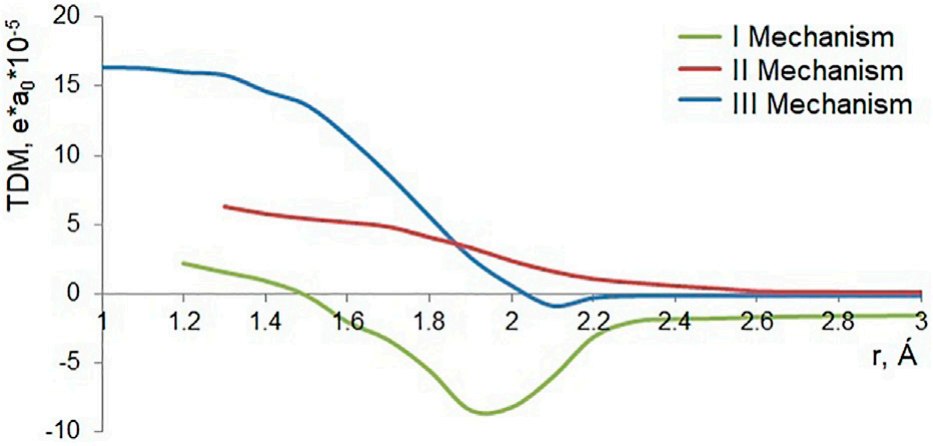
Transition dipole moment contributions of the $1^{3}\Sigma_{g}^{-}\to {\;A}^{3}\Sigma_{u}^{+}$ band system of the N_2_ molecule according to three mechanisms shown schematically in Figure 3.

For the studied transition $1^{3}\Sigma_{g}^{-}\to {\;A}^{3}\Sigma_{u}^{+}$ , only the first “I SOC” mechanism provides an essential sign change with the internuclear distance (Figure 4). In the FC region, no big cancellations of different sign contributions are shown. The deteriorating “I SOC” mechanism is rather weak in the FC region 1.28–1.62 Å. For the most intense 0–7 vibronic band, the calculated EDTM is equal to 1.41×10^−4^ ea_0_, which corresponds to the radiative rate constant of 2.48 s^−1^. The total radiative lifetime of the zero vibrational sublevel of the $1^{3}\Sigma_{g}^{-}$ state is estimated as 0.34 s.

The $1^{3}\Sigma_{g}^{-}$ state can degrade much faster in the allowed T–T transitions (for example, through the $1^{3}\Sigma_{g}^{-}\to {\;B^{\prime }}^{3}\Sigma_{u}^{-}$ emission). Thus, our estimation of the emissive $1^{3}\Sigma_{g}^{-}\to {\;A}^{3}\Sigma_{u}^{+}$ transition is definitely negative. However, in absorption, the same $A^{3}\Sigma_{u}^{+}\left(v^{\prime \prime }=7\right)\;\to {\;1}^{3}\Sigma_{g}^{-}\left(v^{\prime }=0\right)$ transition can be observable since the calculated oscillator strength (f_7–0_ = 2.23*10^−9^) can be measured by modern techniques.

It is, at this point, relevant to estimate the other EDSR-forbidden inter-combination B^3^Π_g_ → *X* ^1^Σ_g_^+^ transition of nitrogen (Wilkinson system) (Lofthus. and Krupenie, 1977), which so far has not been calculated by quantum chemical methods. This is a magnetic dipole transition that borrows intensity from the magnetic singlet–singlet counterpart a^1^Π_g_→*X* ^1^Σ_g_^+^ (Lofthus. and Krupenie, 1977).

## Calculations of magnetic and electric quadrupole transition intensity

**FIGURE 5:**
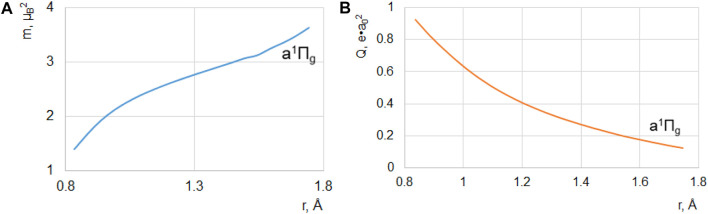
**(A)** Square of magnetic dipole moment m_x_^2^+m_y_^2^ (μ_B_ is the Bohr magneton) and **(B)** electric quadrupole moment of the a^1^Π_g_–X^1^Σ_g_^+^ transition in the N_2_ molecule (both in a. u.).

The Lyman–Birge–Hopfield (LBH) band system (*a* ^1^Π_g_→*X* ^1^Σ_g_^+^) of the N_2_ molecule has been carefully studied in measurements of cascade-induced UV radiation to determine the intensity of this emission (Lofthus. and Krupenie, 1977). The LBH band has readily been seen in absorption as well as in emission though it is EDSR-forbidden by parity selection. Its magnetic and quadrupole transition moments are provided in Figure 5. They are calculated here at the level of the time-dependent density functional theory (TD DFT) using the B3LYP functional and 6-311G++(*d, p*) basis set with the Gaussian-09 package (Frisch et al., 2010). We have studied 40 singlet states and triplet excited states of N_2_ in the region 0.8–1.8 Å of the *r* distances. For the longer N–N bonds, the TD DFT approach produces untrustworthy PECs and cannot reproduce the proper dissociation limits. But for short *r* distances, all potential energy curves are quite reasonable and qualitatively reproduce MRCI results (Dahl and Oddershede, 1986; Qin et al., 2019). This DFT method provides equilibrium bond lengths of 1.205 and 1.598 Å for the triplet (B^3^Π_g_) and quintet (A′^5^Σ_g_^+^) states of nitrogen, respectively. The latter is more realistic (Hochlaf et al., 2010a), whereas the former *r*_*e*_ value deviates slightly from the experimental value of 1.213 Å (Lofthus. and Krupenie, 1977).

**FIGURE 6:**
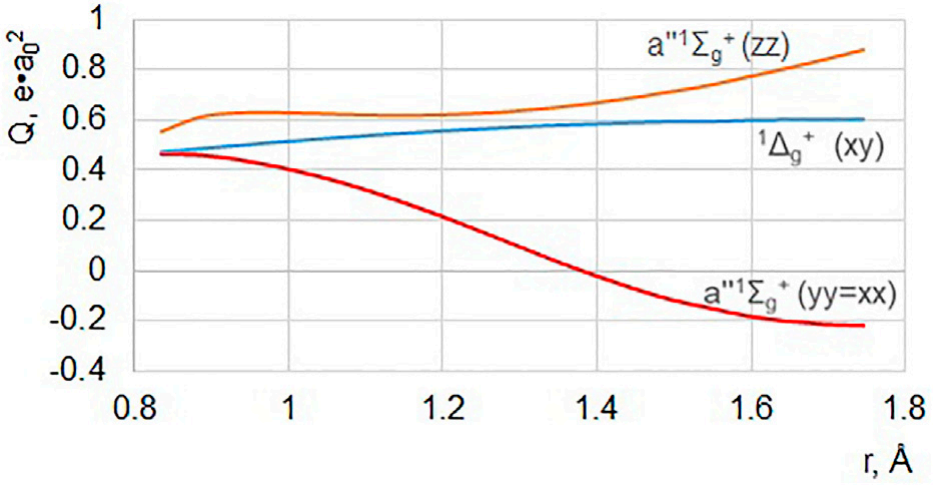
Electric quadrupole moment of the *a”* ^1^Σ_g_^+^–*X* ^1^Σ_g_^+^ transition in the N_2_ molecule for all allowed components of the quadrupole tensor operator. The far-UV transition ^1^Δ_g_– X^1^Σ_g_^+^ for the Q_XY_ = Q_YX_ quadrupole tensor components are also presented (both in a. u.).

A similar approach has been successfully used for the permanent quadrupole moment calculations in N_2_ (Dahl and Oddershede, 1986). In addition to the LBH system, some other EDSR-forbidden bands are also calculated as quadrupole transitions, as shown in Figure 6. The Dressler–Lutz *a"* ^1^Σ_g_^+^–X^1^Σ_g_^+^ quadrupole transition at 101 nm as well as the far-UV transition *z* ^1^Δ_g_–*X* ^1^Σ_g_^+^ (Figure 6) are calculated for the first time.

The growth of magnetic strength of the *a* ^1^Π_g_→*X* ^1^Σ_g_^+^ transition (Figure 5A) and the decrease of its quadrupole moment are notable (Figure 5B). The a″Σ 1g+− *X* ^1^Σ_g_^+^ transition moment represents a complicated tensor with *r*-dependent anisotropy (Figure 6).

In the FC region (1.1–1.3 Å), our results in Figure 5 well coincide with the calculations of Dahl and Oddershede, (1986) using the random phase approximation (RPA). The magnetic dipole transition moment (MDTM) of the LBH system (Figure 5A) increases with *r*, showing a trend of saturation at *r* = 1.3 Å, whereas the electric quadrupole transition moment (EQTM) decreases along the whole *r* range. Accounting for experimental FC factors and transition frequencies, we have obtained the radiative lifetime for the 0–0 vibronic transition of the LBH system equal to 65 μs in a reasonable agreement with experimental values in the interval 80–120 μs (Lofthus. and Krupenie, 1977; Dahl and Oddershede, 1986). The calculated magnetic to quadrupole intensity ratio (m/eq) is equal to 92%, whereas experimental data are in the range of 67%–96% interval (Dahl and Oddershede, 1986). Emission from the higher vibrational levels has a lower probability of qualitative agreement with observations (Lofthus. and Krupenie, 1977; Dahl and Oddershede, 1986). At the same time, we cannot accept the idea that the *a* ^1^Π_g_ state can decay solely into the *X* ^1^Σ_g_^+^ ground state (Dahl and Oddershede, 1986). From Figure 2, one can see that the infrared *a* ^1^Π_g_→*a’* ^1^Σ_u_^−^ emission is possible; its electric dipole transition moment is equal to 0.2 ea_0_ (Qin et al., 2019) using the *r*-centroid approach corresponding to the radiative lifetime for the 0–0 band of τ_r_ = 9 ms (FC factor is 0.219). We have also estimated a new quadrupole transition *a* ^1^Π_g_→*B* ^3^Π_g,1_. Accounting for SOC, in Eq. 8, this transition moment origins in the difference in the permanent quadrupole moments of these two states: Q (*B* ^3^Π_g_) = 0.59 ea_0_^2^ and Q (*a* ^1^Π_g_) = 0.48 ea_0_^2^. This difference is small as well as the quadrupole moment of transition *a→B* (4.9*10^–4^ ea_0_^2^), but in principle, we could not disregard branching emission into other lower lying triplet states (*B′, W*, and *A*) in the calculation of the radiative lifetime of the LBH system. These S–T transitions are allowed in the EDSR approach with an account of spin–orbit coupling perturbation. Thus, we consider it more appropriate to present also the oscillator strength for the Lyman–Birge–Hopfield 0–0 band *a* ^1^Π_g_ ← *X* ^1^Σ_g_^+^ in absorption: f_0–0_ = 7.24×10^−6^.

The Dressler–Lutz *a"* ^1^Σ_g_^+^–*X* ^1^Σ_g_^+^ quadrupole transition in the far-UV absorption region (101 nm) is of the Rydberg type (Lofthus. and Krupenie, 1977); it is well reproduced by our TD DFT calculations. The triplet counterpart of the *a"* ^1^Σ_g_^+^ state is the known *E* ^3^Σ_g_^+^ Rydberg term, which was discussed previously when presenting our calculations of the Herman–Kaplan system ${(E}^{3}\Sigma_{g}^{+}-A^{3}\Sigma_{u}^{+}$ transition). The Dressler–Lutz *a"* ^1^Σ_g_^+^←*X* ^1^Σ_g_^+^ band was observed in absorption at high pressure, and its intensity is mainly induced by collisions (Lofthus. and Krupenie, 1977). In this aspect, it is similar to the quadrupole Noxon band of O_2_, which is very sensitive to collision-induced intensity enhancement (Minaev et al., 1994). Both ^1^Σ_g_^+^ states have similar *r*_*e*_ distance (about 1.1 Å) and FC factor close to unit. The calculated oscillator strength of the 0–0 band of the quadrupole *a"* ^1^Σ_g_^+^ ← *X* ^1^Σ_g_^+^ transition in nitrogen is equal to 1.5·10^−7^, and it is detectable even at low pressure.

Now, we can estimate the probability of the latter triplet–singlet *B* ^3^Π_g_←*X* ^1^Σ_g_^+^ transition of the nitrogen molecule which, being strictly forbidden by ED selection, has not been included in previous calculations (Minaev et al., 1995). This Wilkinson band borrows intensity from the LBH band system (*a* ^1^Π_g_←*X* ^1^Σ_g_^+^) of the N_2_ molecule because of the relatively strong spin–orbit coupling

$${<B}^{3}\Pi_{g,1}|H_{so}^{x,y}{|\;a}^{1}\Pi_{g}{>=41.4\;cm}^{-1},$$ (8)

at the *r*_*e*_ distance and small energy gap between the *B–a* states. Only the Ω = 1 spin sublevel of the triplet *B* ^3^Π_g,1_ state is active in the Wilkinson band absorption, and its rotational structure supports the magnetic transition nature (Lofthus. and Krupenie, 1977). The SOC of Eq. 8 and m_1_ magnetic moment (Figure 5A) provide the largest contribution (98.6%) to the *B* ^3^Π_g_←*X* ^1^Σ_g_^+^ transition intensity. The other *k* ^1^Π_g_ state (1π_u_→3σ_u_) shows a smaller magnetic moment for the *k* ^1^Π_g_–*X* transition (m = 0.085 μ_B_) and a much smaller SOC counterpart at the *B* state equilibrium. Although both parameters increase with *r*, their relative contributions remain rather small. The calculated magnetic transition moment for the 0–0 band of the Wilkinson absorption *B* ^3^Π_g_←*X* ^1^Σ_g_^+^ is equal to 0.0073 μ_B_. It corresponds to the oscillator strength f_0–0_ = 2.54∙10^–10^, and the magnetic intensity remains dominant for this transition. It is not strange that Wilkinson (1962) used an optical path as long as 20 m to detect this band.

Finally, we have estimated the spin-induced magnetic dipole moment for a new ${B^{\prime }}^{3}\Sigma_{u}^{-}-A^{3}\Sigma_{u}^{+}$ transition of the N_2_ molecule. According to Eq. 3, the perturbed *A* state has a small *B’* state admixture for the *M*_*s*_ = ±1 sublevels: |AΣu,1+3〉+CB′,A|B′Σu,1−3〉 . Thus, the transition to the next *M*_*s*_ = 0^+^ spin sublevel of the $B^{\prime }$ state ${B^{\prime }}^{3}\Sigma_{u,0+}^{-}\leftarrow \;A^{3}\Sigma_{u,1}^{+}$ can borrow spin-current intensity from the microwave ${B^{\prime }}^{3}\Sigma_{u,0}^{-}-{B^{\prime }}^{3}\Sigma_{u,1}^{-}$ absorption band with the standard spin-magnetic transition moment that equals 2 μ_B_. For the 0–0 absorption band ${B^{\prime }}^{3}\Sigma_{u}^{-}\leftarrow \;A^{3}\Sigma_{u}^{+}$ , we have obtained oscillator strength f = 1.67 . 10^–12^, which is probably possible for detection.

## Conclusion

The presence of nitrogen atoms in the discharge afterglow classifies “active nitrogen” as a free-radical phenomenon. This is relevant to the aurora borealis’ bright light and the yellow–orange Lewis–Rayleigh afterglow in the N_2_ gas discharge. The spectrum consists of several triplet–triplet emission bands of the **1+** and **2** + nitrogen systems (B^3^Π_g_–A^3^Σ_u_^+^ and C^3^Π_u_–B^3^Π_g_ transitions) and the ${B^{\prime }}^{3}\Sigma_{u}^{-}$ →*B* ^3^Π_g_ infrared-visible afterglow system. The wide Wu–Benesh system *B* ^3^Π_g_ = *W* ^3^Δ_u_ is another T–T transition of the afterglow (Lofthus. and Krupenie, 1977). One can see that many triplet states of the nitrogen molecule take part in discharge afterglow together with numerous T–S transitions and S–S cascades. The transitions allowed by the electric dipole selection rule are nowadays accurately calculated by sophisticated *ab initio* methods (Qin et al., 2019) including many T–S vibronic bands induced by SOC perturbation (Minaev et al., 1995). This is important for the kinetic balance of triplet harvesting in discharges and the Earth’s mesosphere and lower thermosphere regions. In the present work, we have calculated the probability of the magnetic and quadrupole Lyman–Berge–Hopfield transition *a* ^1^Π_g_ ← *X* ^1^Σ_g_^+^, which is necessary for the intensity estimation of the Wilkinson *B* ^3^Π_g_ ← *X* ^1^Σ_g_^+^ band (the only unknown intensity of a pure electronic T–S transition at zero pressure).

We have also calculated new transitions, $1^{3}\Sigma_{g}^{-}\leftarrow \;A^{3}\Sigma_{u}^{+}$ and ${B^{\prime }}^{3}\Sigma_{u}^{-}\leftarrow \;A^{3}\Sigma_{u}^{+}$ , that can be observed during absorption. The reason for finding such transitions is that the first excited triplet state $A^{3}\Sigma_{u}^{+}$ of N_2_ possesses a relatively long radiative lifetime (about 2 s). Therefore, it is possible to excite the triplet–triplet transition from the $A^{3}\Sigma_{u}^{+}$ state by two-photon experiments or other methods of flash photolysis in discharge. We know that the Herzberg I transition was discovered in the oxygen molecule as an excitation from the ground state $X^{3}\Sigma_{g}^{-}$ , but in nitrogen, the situation is reversed since the $1^{3}\Sigma_{g}^{-}$ symmetry corresponds to the upper state.

The $1^{3}\Sigma_{g}^{-}$ state, non-observed so far, has an electronic wave function, which is mainly represented by the valence configuration (1π_u_)^2^ (1π_g_)^2^ in a form similar to a quintet $A\prime^{5}\Sigma_{g}^{+}$ state. The quintet–triplet $A\prime^{5}\Sigma_{g}^{+}$ – $A^{3}\Sigma_{u}^{+}$ transition, also induced by SOC in the electric dipole approach, is the most intense among all studied intercombinations. The spin-induced ${B^{\prime }}^{3}\Sigma_{u}^{-}\leftarrow \;A^{3}\Sigma_{u}^{+}$ transition in the visible region is interesting since it is rather unique in magnetic-origin borrowing intensity from the electron spin resonance in the ${B^{\prime }}^{3}\Sigma_{u}^{-}$ state. The transition intensity could be sensitive to the external magnetic field in solid nitrogen. The ${B^{\prime }}^{3}\Sigma_{u}^{-}\leftarrow \;A^{3}\Sigma_{u}^{+}$ band in N_2_ has common features with the visible A-band of molecular oxygen (Minaev et al., 1994; Minaev and Minaeva, 2001).

Thus, we have noted many important comparable features in N_2_ and O_2_ spectra and also calculated for the first time the intensity of the predicted forbidden transitions including some magnetic dipole and quadruple S–S transitions in the nitrogen molecule. The main new predicted results are summarized in the following table.

**Table:** 

Transition	Absorption		Emission	Wavelength
$1^{3}\Sigma_{g}^{-}\to {\;A}^{3}\Sigma_{u}^{+}$	f_0,0_ = 5.2 10^–12^	f_0,7_ = 2.2 10^–9^	τ = 0.34 s	λ_0,7_ = 328 nm
${A^{\prime }}^{5}\Sigma_{g}^{+}–A^{3}\Sigma_{u}^{+}$	f_0,0_ = 4.9 10^–8^	f_0,6_ = 2.0 10^–4^	τ = 8.23 μs	λ_0,6_ = 598 nm
${B^{\prime }}^{3}\Sigma_{u}^{-}\leftarrow A^{3}\Sigma_{u}^{+}$	f_0,0_ = 1.6 10^–12^	Overlapped by 1 + band	τ = 3750 s	λ = 620 nm

## Data Availability

The raw data supporting the conclusion of this article will be made available by the authors, without undue reservation.
